# The effect of ageing on fMRI: Correction for the confounding effects of vascular reactivity evaluated by joint fMRI and MEG in 335 adults

**DOI:** 10.1002/hbm.22768

**Published:** 2015-02-27

**Authors:** Kamen A. Tsvetanov, Richard N. A. Henson, Lorraine K. Tyler, Simon W. Davis, Meredith A. Shafto, Jason R. Taylor, Nitin Williams, Cam‐CAN, James B. Rowe

**Affiliations:** ^1^ Centre for Speech, Language and the Brain, Department of Psychology University of Cambridge Cambridge United Kingdom; ^2^ Medical Research Council Cognition and Brain Sciences Unit Cambridge United Kingdom; ^3^ School of Psychological Sciences, University of Manchester Manchester United Kingdom; ^4^ Cambridge Centre for Ageing and Neuroscience (Cam‐CAN) University of Cambridge and MRC Cognition and Brain Sciences Unit Cambridge United Kingdom; ^5^ Department of Clinical Neurosciences University of Cambridge Cambridge United Kingdom

**Keywords:** aging, cerebral vascular reactivity, signal variability, scaling, resting state, fluctuation amplitude

## Abstract

In functional magnetic resonance imaging (fMRI) research one is typically interested in neural activity. However, the blood‐oxygenation level‐dependent (BOLD) signal is a composite of both neural and vascular activity. As factors such as age or medication may alter vascular function, it is essential to account for changes in neurovascular coupling when investigating neurocognitive functioning with fMRI. The resting‐state fluctuation amplitude (RSFA) in the fMRI signal (rsfMRI) has been proposed as an index of vascular reactivity. The RSFA compares favourably with other techniques such as breath‐hold and hypercapnia, but the latter are more difficult to perform in some populations, such as older adults. The RSFA is therefore a candidate for use in adjusting for age‐related changes in vascular reactivity in fMRI studies. The use of RSFA is predicated on its sensitivity to vascular rather than neural factors; however, the extent to which each of these factors contributes to RSFA remains to be characterized. The present work addressed these issues by comparing RSFA (i.e., rsfMRI variability) to proxy measures of (i) cardiovascular function in terms of heart rate (HR) and heart rate variability (HRV) and (ii) neural activity in terms of resting state magnetoencephalography (rsMEG). We derived summary scores of RSFA, a sensorimotor task BOLD activation, cardiovascular function and rsMEG variability for 335 healthy older adults in the population‐based Cambridge Centre for Ageing and Neuroscience cohort (Cam‐CAN; http://www.cam-can.com). Mediation analysis revealed that the effects of ageing on RSFA were significantly mediated by vascular factors, but importantly not by the variability in neuronal activity. Furthermore, the converse effects of ageing on the rsMEG variability were not mediated by vascular factors. We then examined the effect of RSFA scaling of task‐based BOLD in the sensorimotor task. The scaling analysis revealed that much of the effects of age on task‐based activation studies with fMRI do not survive correction for changes in vascular reactivity, and are likely to have been overestimated in previous fMRI studies of ageing. The results from the mediation analysis demonstrate that RSFA is modulated by measures of vascular function and is not driven solely by changes in the variance of neural activity. Based on these findings we propose that the RSFA scaling method is articularly useful in large scale and longitudinal neuroimaging studies of ageing, or with frail participants, where alternative measures of vascular reactivity are impractical. *Hum Brain Mapp 36:2248–2269, 2015*. © **2015 The Authors Human Brain Mapping Published by Wiley Periodicals, Inc.**

## INTRODUCTION

With the global demographic shift towards an older population, and the impact that age can have on cognition and dementia, there is a pressing need to understand the neurobiology of cognitive ageing. Functional brain imaging with functional magnetic resonance imaging (fMRI) has many advantages as a tool to study ageing and much of the current theoretical understanding of neurocognitive ageing has drawn on blood‐oxygenation level‐dependent (BOLD) based fMRI, linked to the cognitive ontologies from cognitive neuroscience. However, the BOLD signal is not a direct measure of neural activity, but is a complex convolution of neural and vascular signals (Logothetis, 2008). There is a risk therefore that fMRI studies that have been interpreted in terms of neural or neurocognitive change with age, instead reflect the effects of ageing on vasculature and neurovascular coupling.

Ageing affects the neural and vascular components of the BOLD fMRI signal differentially (D'Esposito et al., 2003; Hutchison et al., 2012, 2013). Without correction for vascular effects, the attribution of age‐related differences in task‐related BOLD signal to neural functioning may be overestimated (Liu et al., 2013). Several methods enable the estimation of vascular differences, including hypercapnia (Bright et al., 2009; Liu et al., 2013; Lu et al., 2011; Yezhuvath et al., 2009), breath‐holding induced hypercapnia (Handwerker et al., 2007; Mayhew et al., 2010a; Riecker et al., 2003; Thomason et al., 2005, 2007), cerebral blood flow and venous oxygenation (Liau and Liu, 2009; Lu et al., 2010; Restom et al., 2007). However, these are impractical in larger cohort studies of ageing, and may be poorly tolerated by older adults (for a review, see Liu et al., 2012). Additionally, hypercapnic challenge may not be entirely neuronally neutral: for example, participant awareness of the challenge might produce confounding effects in this normalization approach (Hall et al., 2011), suggesting a potential advantage for scaling approaches that offer “task‐free” estimates of vascular reactivity.

Kannurpatti and Biswal (2008) proposed the use of resting state (“task‐free”) fMRI to derive a measure of vascular reactivity. In their work, Kannurpatti and Biswal (2008) derived resting‐state fluctuation amplitude (RSFA) maps from the BOLD signal variance on a whole‐brain voxel‐wise manner and demonstrated a high correspondence of intra‐ and interparticipant variance (over 80%) between RSFA and the response to hypercapnic challenge. Since RSFA reflects naturally occurring variations in cardiac rhythm and respiratory rate and depth (Birn et al., 2006; Chang et al., 2013; Golestani et al., 2015; Wise et al., 2004), this approach approximates breath‐hold normalization. In addition, fluctuations in the vascular system, for example, heart rate variability (HRV), are a significant source of physiological noise in the resting‐state BOLD signal variance (Shmueli et al., 2007), independent from other sources of physiological noise (Chang et al., 2009, 2013). High and low frequency fluctuations of heart rate (HRV) decrease with age, reflecting asymptomatic changes in the regulation of the autonomic nervous system and age‐related changes in cerebrovascular regulation (Cheitlin, 2003; Reardon and Malik, 1996; Varadhan et al., 2009; Zulfiqar et al., 2010).

The RSFA method assumes that age‐related differences in RSFA reflect purely vascular signals (Behzadi and Liu, 2005) and does not capture sources of neural origin. However, it is possible that age‐related differences in resting neural reactivity also contribute to rsfMRI signal changes with age (Dustman et al., 1993, 1999; McIntosh et al., 2013; Pierce et al., 2000). Electroencephalographic (EEG) and magnetoencephalographic (MEG) recordings can be used to assess neural activity without vascular confounds. Cross‐sectional studies have shown associations between resting state EEG/MEG power with resting state BOLD fluctuations (Brookes et al., 2011; De Munck et al., 2008; Goldman et al., 2002; Gonçalves et al., 2006; Laufs et al., 2006; Mayhew et al., 2010b, 2013; Moosmann et al., 2003; Neuner et al., 2014) and the resting state energy metabolism (Alper et al., 2006; Larson et al., 1998; Oakes et al., 2004).

The present work examined the use of rsfMRI measures (i.e., RSFA) to correct for the vascular effects of age when studying task‐induced BOLD fMRI responses. First, we characterized age differences in RSFA and determined the effects of RSFA scaling on age‐dependent fMRI‐BOLD signal change in a sensorimotor task. We predicted (i) that spatial patterns of age differences in RSFA would be similar to those reported by other calibrating techniques; and (ii) that the use of RSFA scaling would reduce the apparent effects of ageing on BOLD‐fMRI responses, by revealing the underlying effects of age on neural response.

Importantly, given the unique combination of fMRI, MEG and physiological data from the large number of participants afforded by the Cam‐CAN dataset, we were able to validate the use of RSFA as a scaling factor. We did this by using mediation models to determine the extent to which age differences in RSFA were dependent on vascular and neural sources of signal fluctuations. In the first two mediation models, we examined the dependency of interindividual differences in RSFA with measures of vascular performance (i.e., HRV) and neural activity (i.e., resting state MEG signal variability across time of standard frequency bands). We predicted there would be significant mediating effects of HRV, but not of neural variability, on the age differences in BOLD response variability. In a final confirmatory mediation analysis we determined whether or not HRV mediates the effect of ageing on neural variability.

## METHODS

### Participants

A healthy, population‐based sample was collected as part of the Cambridge Centre Ageing and Neuroscience (Cam‐CAN). Ethical approval for the study was obtained from the Cambridgeshire 2 (now East of England ‐ Cambridge Central) Research Ethics Committee. Participants gave written informed consent. Exclusion criteria included poor vision (below 20/50 on Snellen test; Snellen, 1862) and poor hearing (failing to hear 35 dB at 1000 Hz in both ears), ongoing or serious past drug abuse as assessed by the Drug Abuse Screening Test (DAST‐20; Skinner, 1982), significant psychiatric disorder (e.g., schizophrenia, bipolar disorder, personality disorder) or neurological disease (e.g., known stroke, epilepsy, traumatic brain injury). At an initial home assessment, all participants completed the Mini‐Mental State Examination (MMSE; Folstein et al., 1975) with scores within the range of normal cognitive ability (see Table 1). Handedness was assessed using Edinburgh Handedness Inventory (Oldfield, 1971). Participants then attended MRI (structural and functional) and MEG scan sessions on two different days, separated by approximately 8 weeks. In total, 335 participants had good quality full datasets required for all analysis (e.g., T1‐image, sensorimotor‐task fMRI, fMRI‐, MEG‐, ECG‐, and pulse oximeter resting state recordings, see below).

**Table 1:** Participants' demographic information (% of age decile given in round brackets)

	Decile							Statistical tests^a^	
	1	2	3	4	5	6	7	X^2^ or F‐test	*P*‐value
Age range [years]	18–27	28–37	38–47	48–57	58–67	68–77	78–87		
Gender								3.64	0.725
Men	14 (60.9)	32 (59.3)	35 (59.3)	29 (52.7)	32 (49.2)	22 (45.8)	17 (54.8)		
Women	9 (39.1)	22 (40.7)	24 (40.7)	26 (47.3)	33 (50.8)	26 (54.2)	14 (45.2)		
Handedness^b^								0.80	0.572
Mean/ SD	80/41	78/50	74/53	85/40	78/52	88/37	91/31		
Range [min/max]	−100/100	−100/100	−100/100	−100/100	−100/100	−90/100	−70/100		
MMSE								6.76	<0.001
Mean/SD	29.5/1.1	29.3/1.2	28.9/1.2	29.3/0.9	29/1.2	28.2/1.5	28.4/1.3		
Range [min/max]	25/30	25/30	26/30	27/30	26/30	25/30	25/30		

a Statistical test to indicate whether demographics vary between different deciles

b Higher scores indicate greater right‐hand preference

### fMRI Acquisition and Preprocessing

Imaging data were acquired using a 3T Siemens TIM Trio System at the MRC Cognition Brain Sciences Unit. A 3D‐structural MRI was acquired on each participant using T1‐weighted sequence (Generalized Autocalibrating Partially Parallel Acquisition (GRAPPA); repetition time (TR) = 2,250 ms; echo time (TE) = 2.99 ms; inversion time (TI) = 900 ms; flip angle *α* = 9°; field of view (FOV) = 256 × 240 × 192 mm^3^; resolution = 1 mm isotropic; accelerated factor = 2) with acquisition time of 4 min and 32 s.

**Figure 1:**
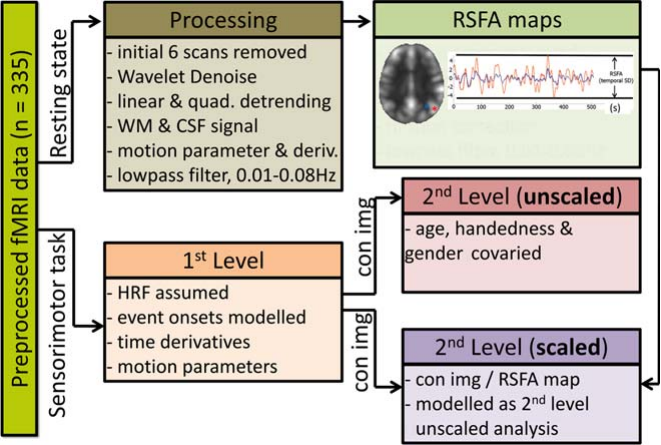
Processing of resting state fMRI data and scaled/unscaled sensorimotor task fMRI BOLD data. [Color figure can be viewed in the online issue, which is available at http://wileyonlinelibrary.com.]

For resting state fMRI measurements, echo‐planar imaging (EPI) data of 261 volumes were acquired with 32 slices (sequential descending order), slice thickness of 3.7 mm with a slice gap of 20% (whole brain coverage; TR = 1,970 ms; TE = 30 ms; flip angle *α* = 78°; FOV = 192 × 192 mm^2^; resolution = 3 × 3 × 4.44 mm^3^) during 8 min and 40 s. Participants were instructed to lie still with their eyes closed. The initial six volumes were discarded to allow for T1 equilibration. The preprocessing and statistical analyses were carried out in SPM 12 (http://www.fil.ion.ucl.ac.uk/spm). The preprocessing steps included (i) spatial realignment to correct for head movement and movement by distortion interactions, (ii) temporal realignment of all slices to the middle slice, (iii) coregistration of the EPI to the participant's T1 anatomical scan, (iv) unified‐segmentation‐normalization to normalize the T1 image to the MNI template, the parameters of which were then applied to the functional data and (v) spatial smoothing with an FWHM of 8 mm to meet the lattice assumption of random field theory and account for residual interparticipant structural variability. Further processing procedures of the resting state time series for estimation of RSFA were performed using a combination of the following steps (see Fig. 1 for a schematic representation): The first step was to apply a data‐driven approach for removing motion artefacts from fMRI data (Patel et al., 2014). We further included linear and quadratic detrending of the fMRI signal, covarying out white matter (WM) and cerebrospinal fluid (CSF) signal, and regression of the motion parameters and their first derivative. WM and CSF signals were estimated for each volume from the mean value of WM and CSF masks derived by thresholding SPM's tissue probability maps at 0.75. The resting data were bandpass‐filtered (0.01–0.08 Hz) and the standard deviation (SD) of these filtered time‐series was calculated on a voxel‐wise basis to define RSFA. The bandpass filter was chosen for consistency with previous studies, which sought to maximize the dependency of rsBOLD signal fluctuations on physiological causes (Chang et al., 2009; Di et al., 2012; Glover et al., 2000; Kannurpatti and Biswal, 2008).

We also acquired task‐induced BOLD data from a sensorimotor paradigm. In this task, 120 trials comprised of a bilateral black and white checkerboards and binaural tone stimulation at one of three frequencies (300, 600, or 1,200 Hz, equal number of trials pseudorandomly ordered) with a duration of 34 and 300 ms, respectively. In addition, eight unimodal (four auditory only and four visual only) stimuli were randomly distributed throughout the whole run. For each trial, participants responded by pressing a button with their right index finger if they hear or see any stimuli. In addition, null events, comprised of a fixation cross in the centre of the screen, were interdispersed with events of interest. To maximize efficiency, real and null events were ordered using a 255‐length m‐sequence (Buračas and Boynton, 2002), with *m* = 2 and minimal stimulus onset asynchrony (SOA) of 1.97 s (resulting in SOAs ranging from ∼2–26 s). Scanning parameters and preprocessing steps were the same as for resting state data. Event‐related fMRI analysis for the sensorimotor‐task used a canonical haemodynamic response function (HRF) and its temporal and dispersion derivatives (Friston et al., 1998) to model the haemodynamic activity to bilateral stimulation trials. Regressors of no interest included catch trials, error trials, head motion and harmonic regressors that capture low frequency changes (1/128 Hz) in the signal typically associated with scanner drift and physiological noise. Second level, random effects analyses were performed on a GLM that included the linear effect of age and a constant term, with statistical contrasts of these effects thresholded after voxel‐wise correction of family‐wise error (FWE) at *P* < 0.05.

### Haemodynamic Scaling of Task‐Evoked BOLD Activation

We compared the effects of ageing on task‐induced BOLD activity before and after scaling by the RSFA. Scaling entailed dividing the parameter estimate for the canonical HRF fit to the mean evoked response in the sensorimotor‐task for each participant by their RSFA value at the same voxel. In a group‐level analysis, we estimated the effects of ageing using separate GLMs for unscaled (before RSFA scaling) and scaled (after RSFA scaling) activation maps (see Fig. 1). To formally address the differences between analyses with scaled‐ and unscaled‐BOLD signal, we constructed an additional second‐level GLM analysis with activation maps estimated from the difference between scaled‐ and unscaled‐BOLD maps.

### Resting State MEG Acquisition and Preprocessing

MEG data were acquired approximately 8 weeks before or after fMRI acquisition, in a magenetically shielded room using a Vectorview system (Elekta Neuromag, Helsinki) with 102 magnetometers and 204 orthogonal planar gradiometers. Vertical and horizontal eye movement was recorded using paired electrooculography (EOG) electrodes. Cardiac rhythm was monitored using paired electrocardiogram (ECG) electrodes – one on the right scapula and one on the left lower rib. Head movements were continuously monitored with respect to the MEG sensors using five head‐position indicator (HPI) coils. Position of the HPI coils, of approximately 100 “head points” across the scalp, and of three anatomical fiducials (the nasion and left and right preauricular points) were recorded using a 3D digitizer (Fastrak Polhemus, Colchester, VA). Data were sampled at 1 kHz with a band‐pass filter of 0.03–330 Hz. Participants were in seated position with eyes closed for approximately 9 min. The initial 30‐s recording interval was removed from further analysis to allow a steady state of the recorded signal, resulting in 504321 samples for each individual, and provide a resting state recording with a duration similar to the duration of the fMRI resting state recording (∼8.5 min).

Preprocessing of the MEG continuous data was performed with MaxFilter 2.2 (Elekta Neuromag Oy, Helsinki, Finland). This uses spherical basis functions to remove environmental noise based on magnetic fields outside a sphere enclosing the sensor array, by rejecting temporally correlated sources inside this sphere but outside a sphere enclosing the brain (so‐called temporal extension of signal space separation (SSS); Taulu et al., 2005), with a correlation threshold of 0.98 and a 10 s sliding window. The same spherical harmonic representation allows movement correction every 200 ms (based on the HPI coils), and to align the data within a common space across participants (as if the centre of their heads were in the same position relative to the sensors), thereby facilitating comparison of data from the same sensors across participants despite their variable true head position. Finally, Maxfilter was also used to detect and reconstruct bad channels, and to notch‐filter the line noise at 50 Hz, its harmonics and the HPI signals (>300 Hz).

To remove residual non‐brain physiological artifacts of ocular or cardiac origin, the data were subjected to independent component analysis (ICA). Each independent component (IC) was then correlated with the recorded vertical EOG, horizontal EEG, and ECG. ICs that showed a high temporal correlation with one of the recorded EOG or ECG channels, and (for those that correlated temporally with EOG channel) a high spatial correlation with pre‐defined template topographies for vertical and horizontal eye‐movement were projected out of the data. The topographies were created by ICA of independent data, where the template topographies corresponded to the spatial part of those ICs that were clearly blinks, horizontal eye‐movements, or cardiac (based on manual inspection of time course, frequency spectrum and topography). We then averaged those spatial components over ∼18 subjects. A high temporal correlation was defined as at least three times the standard deviation of all IC correlations with each EOG/ECG channel; a high spatial correlation was defined as at least two times the standard deviation of all IC correlations with a template topography.

Finally, we wanted to derive a measure of neural variability to relate to the rsfMRI variability. Previous work suggests that Hilbert Envelope signal variability shows linear relationship with raw signal variability of MEG time series (Hall et al., 2013) and correlates with BOLD variability, particularly in the beta band (Brookes et al., 2011; De Pasquale et al., 2010; Luckhoo et al., 2012). To keep our analysis consistent across modality signals, that is, to RSFA, derived from the SD of rsBOLD time series, we calculated SD of resting MEG time series for a number of standard frequency bands (subdelta: 0.01–0.08 Hz; delta: 1–4 Hz; theta: 4–8 Hz; alpha: 8–13 Hz; beta: 13–30 Hz; gamma: 30–80 Hz). We derived these data for the magnetometers only, given their deeper field of view.

### Physiological Recordings and Preprocessing

Cardiac activity data were acquired in parallel to fMRI and MEG recordings using photoplethysmograph/pulse‐oximeter placed on the left index finger of the participant (in the case of fMRI session) and with the ECG (in the case of the MEG session). The sampling frequency for PulseOx data was 50 Hz, while the ECG data was sampled at 1 kHz. Preprocessing of the cardiac data was performed in Matlab (MATLAB 8.1, The MathWorks, Natick, MA, 2013). This included R‐wave 1R waves typically present the largest amplitudes compared to P, Q, S, and T waveforms in the ECG signal. detection using PeakFinder function from MatlabCentral, where optimal identification of peaks was confirmed by visually inspecting raw data. Next, we calculated the time difference between each pair of peaks as a measure of interbeat‐interval (IBI) in milliseconds. To further screen for outlier IBIs which may result from ectopic beats (Aubert et al., 1999; Tarvainen, 2004), peak‐detection errors and/or noisy segments in the recording, we adopted an iterated outlier estimation procedure (Selst and Jolicoeur, 1994). In particular, the cut‐off for the first pass was determined from the mean and SD from the normal distribution of the whole IBI sample after temporary excluding the most extreme observation in the sample. Values that exceeded three standard deviations were removed (classified as outliers) and a new cut‐off from the resulting sample was estimated. This procedure was iterated until no further outliers were detected. The resulting sample of IBI values from the last iteration was used for further analysis to derive measures of cardiac function as follows.

Estimation of mean heart rate (HR) was based on the mean number of IBIs within each 60‐s interval during the recording. To estimate the HRV, we used the frequency‐domain information of IBI, which provides a measure of low‐ and high‐ frequency components of the HRV, relative to time‐domain alternatives, for example, the root mean squared difference of successive intervals (RMSSD) which pertains mainly to high‐frequency dynamics of HRV (Malik et al., 1996). We therefore calculated low‐frequency (0.05–0.15 Hz; LF‐HRV) and high‐frequency (0.15–0.4 Hz; HF‐HRV) power of the IBI time‐series (the entire resting state acquisition) in the frequency domain using HRV Analysis Software (Ramshur, 2010).

### Data Reduction

**Figure 2:**
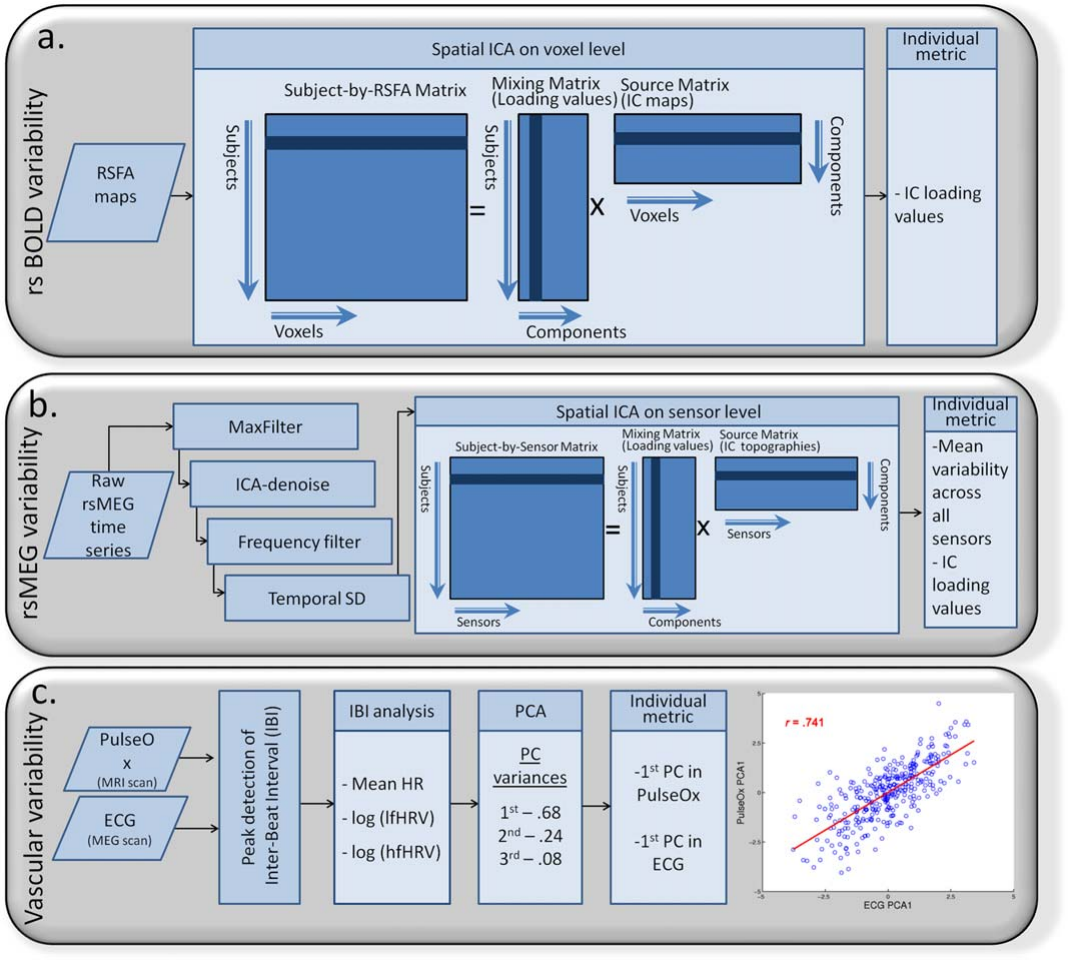
Data reduction of multimodal datasets to derive individual metrics. **a**) spatial ICA to create RSFA maps in which a participant‐by‐voxel matrix was decomposed into a mixing matrix and source matrix. **b**) preprocessing of resting‐state MEG timeseries data to derive rsMEG variability at each sensor, and spatial ICA across MEG sensors (102 magnetometers). **c**) Estimation of vascular health indices from pulseoximetry (PulseOx) and ECG data by decomposing the shared variance between mean HR, low‐ and high‐frequency HRV using PCA. Right of panel c, a scatter plot demonstrating high predictability between ECG‐ and PulseOx‐based PCA1 loading values. [Color figure can be viewed in the online issue, which is available at http://wileyonlinelibrary.com.]

As the datasets of interest (RSFA, rsMEG, and ECG/PulseOx measures) were from different modalities, we computed summary measures for each of these metrics to allow statistical analysis without excessive risk of multiple statistical comparisons. We started by calculating the SD across time for fMRI/MEG resting state time series data (RSFA and rsMEG variability, respectively). We then used ICA across participants to derive spatial patterns of RSFA across voxels and rsMEG variability across sensors expressed by the group in a small number of ICs. The number of ICs was estimated using PCA with a minimum description length (MDL) criterion (Hui et al., 2011; Li et al., 2007; Rissanen, 1978). As a proxy of vascular health, we used PCA to derive a latent variable from a set of measures related to cardiac function derived from the resting HR signal. Figure 2 gives a schematic presentation of the steps to derive summary measures.

#### Indices of RSFA using independent component analysis

RSFA maps were decomposed in a set of spatially independent sources using Source Based Morphometry toolbox (SBM v1.0b, Xu et al., 2009), part of the Group ICA for fMRI Toolbox (GIFT; http://mialab.mrn.org/software/gift). In brief, the fastICA algorithm was applied after the optimal number of sources explaining the variance in the data was identified using PCA with MDL criterion (Li et al., 2007). By combining the PCA and ICA, one can decompose an *n*‐by‐*m* matrix of participants‐by‐voxels into (1) a source matrix that maps independent components (ICs) to voxels (here referred to as “IC maps”), and (2) a mixing matrix that maps ICs to participants. The mixing matrix indicates the degree to which a participant expresses a defined IC, see Figure 2. The IC loading values in the mixing matrix were scaled to standardized values (*Z*‐scores) and used in the subsequent between‐participants analysis with standardized summary measures from other modalities.

#### Indices of rsMEG variability using independent component analysis

Analogously to the RSFA, the number of components of MEG variability was estimated with an MDL criterion and PCA, but for each frequency band separately, followed by ICA using a fastICA algorithm (Gävert et al., 2005). After decomposing the participant‐by‐magnetometer matrix this way, the source matrix described the relationship between components and sensors (i.e., IC topographies) and the mixing matrix described the relationship between participants and components (i.e., IC loading values); see Figure 2.

#### Indices of vascular health using principal component analysis

In the case of the vascular health index, we were interested in obtaining a summary measure characterizing the complexity of the cardiac signal. Previous work suggested that the use of PCA on traditional HRV indices provides a compact summary to study age differences in the HR signal (Varadhan et al., 2009). Therefore, we conducted PCA on the mean HR, LF‐HRV and HF‐HRV to reduce the dimensionality into one latent variable summarizing the largest portion of shared variance, that is, the first principal component (PC1). The analysis was derived separately for the PulseOx and ECG data see Figure 2.

### Mediation Pathway Analysis

The current study sought to dissociate the relationships between (i) RSFA and vascular markers, and (ii) RSFA and neural variability (grand mean of time‐series variability across all sensors for six frequency bands and IC loading values), as part of the assessment of the use of RSFA to correct for the effects of age on BOLD fMRI. Since several measures of interest were likely to be correlated due to shared age‐related variance, we used mediation pathway analysis (Hayes, 2013), to test how ageing affects RSFA via vascular coupling and/or underlying neural activity (see Fig. 8 for further details). The analyses were conducted with the mediation toolbox for Matlab (Wager et al., 2008), using ordinary least squares, three‐variable path models with a bias‐corrected bootstrap test for statistical significance of the indirect path based on 10,000 bootstrap samples. Significance was declared when the 99% confidence interval (CI) excluded zero. We tested three types of mediation models (all models included information about handedness and gender (Dart, 2002; Schank et al., 2007)) as follows:

– Model 1 tested whether HRV mediates the effect of age on RSFA, where age was the independent variable, HRV was the mediator and RSFA was the dependent variable (green colour paths in Fig. 8). Each individual's proxy for HRV was their loading value of the first principal component of the PulseOx data (PC1_PulseOx_), which was acquired simultaneously with the resting state BOLD data. As a summary measure of each individual's RSFA, we used the IC loading value for each of a number of RSFA‐ICs. Only RSFA‐ICs showing ageing differences in the loading values were included in Model 1.
– Model 2 tested whether neural activity at rest (i.e., rsMEG variability) mediated the effects of age on RSFA, where age was the independent variable, rsMEG was the mediator and RSFA was the dependent variable (orange colour paths in Fig. 8). An individual's proxy for neural activity at rest, for each of the six frequency bands, was either their mean rsMEG variability across all sensors, or their IC loading value for those MEG ICs that correlated with age.
– Model 3 tested whether HRV mediated the effect of age on neural variability, where age was the independent variable, HRV was the mediator and rsMEG variability was the dependent variable (blue colour paths in Fig. 8). Here, HRV was based on PC1_ECG_ loading values from the ECG recordings during the resting state MEG acquisition. Model 3 was again run with the mean rsMEG variability across all sensors or IC loading values for those MEG ICs that correlated with age.

## RESULTS

### Spatial Distribution and Age Differences in RSFA

**Figure 3:**
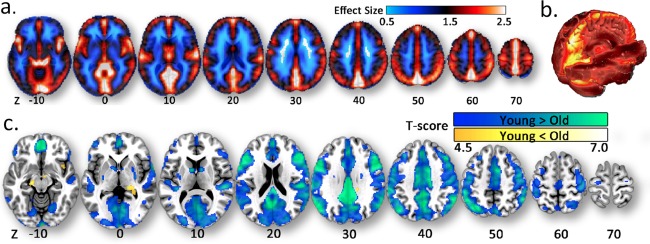
**a**) Average RSFA across all participants overlaid on a representative participant's T1‐image. **b**) Results in a) presented in a rendered view. **c**) Age‐related decreases (cold colours) and increases (warm colours) in RSFA. The threshold was set at *P* <0.05, FWE‐corrected. *Z*‐values refer to the axial slice in MNI space.

Whole group analysis revealed relatively high numerical values of RSFA (relative to the average across the brain) in the frontal orbital, inferior frontal gyrus (IFG), dorsolateral prefrontal cortex (dlPFC), superior frontal cortex, anterior and posterior cingulate, and lateral parietal cortex (see Fig. 3a,b).

With respect to ageing, we observed significant decreases in RSFA as a function of age in the bilateral IFG, bilateral dlPFC, bilateral superior frontal gyrus (SFG), primary visual cortex, cuneus, precuneus, posterior and anterior cingulate, superior temporal gyrus, medial parietal cortex, and lateral parietal cortex (Fig. 3c). Regions in the proximity of ventricles and large vascular vessels showed a significant increase of RSFA values as a function of age.

### Application of RSFA Scaling to BOLD Response to Audiovisual Stimulation

#### Wholegroup BOLD response to audiovisual stimulation

**Figure 4:**
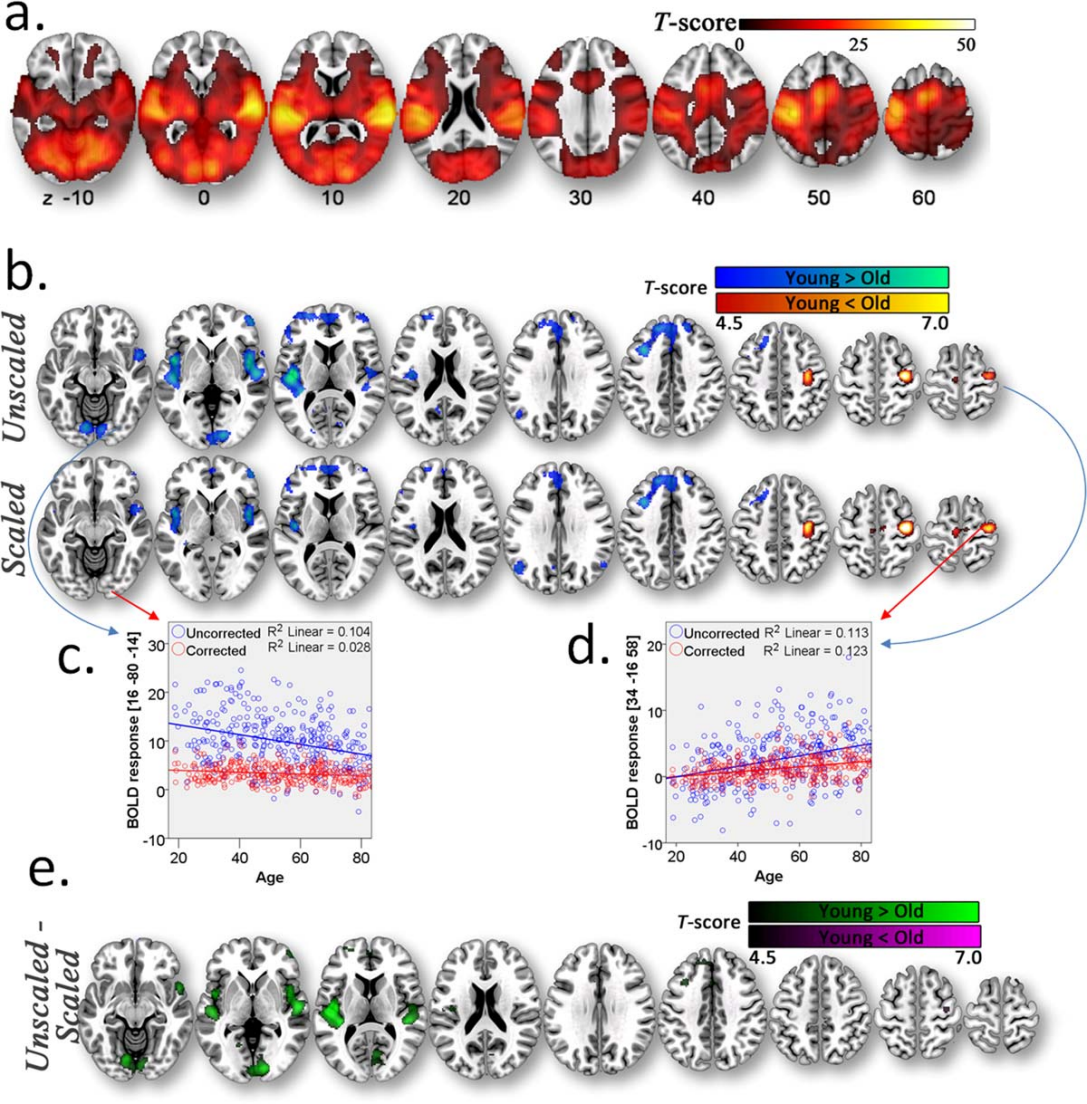
**a**) Group BOLD activity in the primary visual, motor and auditory cortices in the sensorimotor task. **b**) Decreases (cold colours) and increases (warm colours) in unscaled and scaled BOLD response as a function of increasing age. **c**) Decrease in BOLD response as a function of increasing age in the occipital lobe (blue) is abolished after scaling the sensorimotor‐task with RSFA(red), where each point represents an individual. **d**) Age‐related increase in BOLD response in the ipsilateral motor cortex remains after scaled by RSFA. **e**) Apparent age‐related decreases in BOLD response in primary visual and auditory cortices, on the other hand, are no longer present after scaling by RSFA. The threshold was set at *P* <0.05, FWE‐corrected. [Color figure can be viewed in the online issue, which is available at http://wileyonlinelibrary.com.]

GLM analysis revealed that the group BOLD response to audiovisual stimulation versus interstimulus baseline included the primary visual cortex, bilateral auditory cortex, the left motor/sensorimotor cortex, and the supplementary motor area (SMA; Fig. 4a).

#### **Ageing effects on BOLD response to audiovisual stimulation and the effects of haemodynamic scaling with RSFA**

Using linear regression, we explored the effects of ageing on the BOLD response to audiovisual stimulation both with and without scaling by RSFA. Analysis of the data without controlling for RSFA revealed age‐related decreases in the bilateral auditory cortex, the early visual cortex, left lateral parietal cortex, and the anterior cingulate, coupled with age‐related increases in the ipsilateral motor cortex (Fig. 4b*, unscaled*). After RSFA scaling, however, the observed age‐related decreases in the visual areas and extended portion of the auditory areas (Fig. 4b*, scaled*) were abolished (Fig. 4c). Interestingly, age‐related increases in the ipsilateral motor cortex, however, remained significant after RSFA scaling (Fig. 4d).

To formally address the differences between scaled and unscaled analyses, we directly contrasted the regression slopes for scaled versus unscaled BOLD data against age. The results demonstrated that RSFA scaling of the BOLD signal significantly reduced the age effect in the visual and auditory areas (Fig. 4e).

### Validation of RSFA

The use of RSFA as a proxy for vascular reactivity depends on the assumption that age‐related differences in RSFA variability are vascular, that is, do not reflect differences in neural fluctuations at rest. To address this, we examined individual differences in RSFA in relation to measures of (i) vascular health, that is, HR and HRV and (ii) neural activity, that is, rsMEG signal variability of the time course of standard frequency bands.

#### Regional RSFA using ICA

**Figure 5:**
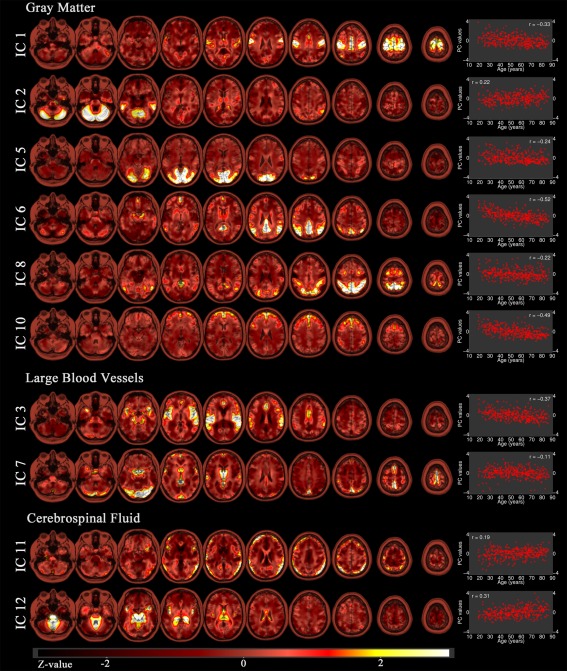
Spatial distribution of 10 (out of 12) independent components (ICs) after ICA across participants of RSFA values at each voxel. ICs that follow gray matter (top panel), or vascular regions (middle panel) or CSF (bottom panel) show age‐dependent differences in their loading values across participants (shown in scatter plots with respective *r* values right of each map). Group‐level spatial maps rendered on a structural template, where intensity values correspond to *Z*‐values (see colour bar). [Color figure can be viewed in the online issue, which is available at http://wileyonlinelibrary.com.]

From ICA of the RSFA data, there were 12 components according to the MDL criterion (Li et al., 2007). Ten ICs showed age‐dependent changes in the expression of the loading values (Fig. 5). The components revealed spatial patterns suggesting origins of signal from (i) gray matter (GM), (ii) vascular etiology, and (iii) CSF. One of the vascular components reflected signals from arterial supply, including the circle of Willis, basilar artery, internal carotid artery, posterior communicating artery, anterior cerebral artery, and middle cerebral artery (see Fig. 5, IC3). A second vascular component reflected signals originating near territories of venous drainage, including superior sagittal sinus, internal cerebral vein, anterior cerebral vein, superior ophthalmic vein, straight sinus, and transverse sinus (see Fig. 5, IC 7).

The age‐sensitive components with spatial distribution within GM included sensory areas (i.e., visual areas, auditory and somatosensory cortical areas, IC 1, and IC 5), cerebellum (IC 2), posterior association areas (i.e., ventrolateral partietal cortex, IC6, and IC8), and anterior association areas (IC 10). These components, except for IC2, were more strongly expressed by young adults (i.e., exhibited an age‐related decrease of IC loading values, see Table 3).

The last two components reflected age‐related increase of RSFA within the ventricles and cerebral aqueduct (IC12), and subarachnoid space (IC11).

#### Spatial distribution and age differences in rsMEG variability

##### Topography of mean rsMEG variability in sensor space

**Figure 6:**
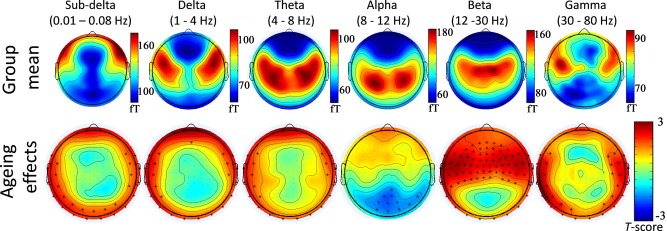
Topography of the mean rsMEG variability for standard frequency bands (top panel) and ageing effects on rsMEG variability (bottom panel) as measured by magnetometers. Age‐related increases and decreases of rsMEG variability are shown in red and blue colours, respectively. Asterisk denotes channels with significant effect of age, at *P* < 0.01. [Color figure can be viewed in the online issue, which is available at http://wileyonlinelibrary.com.]

The topographic distribution of mean rsMEG variability across all participants revealed spatial distributions with largest signal variability in fronto‐temporal regions for the subdelta and delta bands, in superior fronto‐parietal regions for the theta band, in occipito‐parietal regions for the alpha band, in central areas (e.g, motor areas) for the beta band and in fronto‐temporal areas for the gamma band (see Fig. 6).

**Figure 7:**
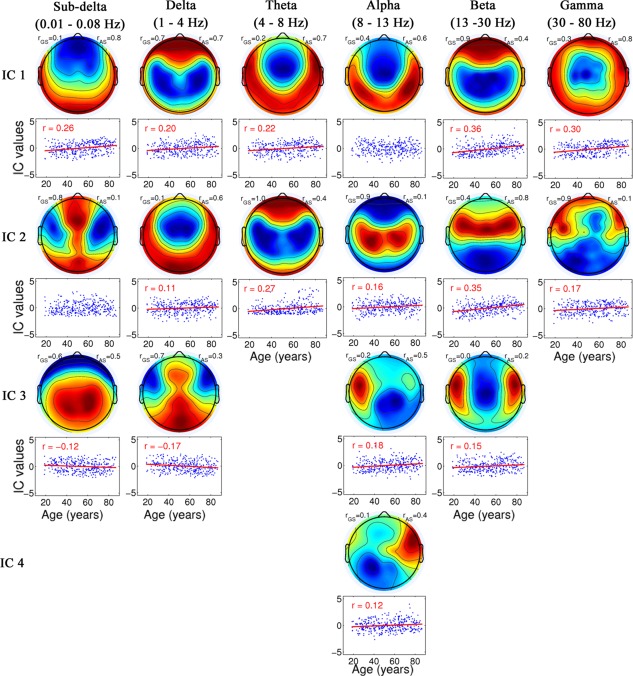
ICA decomposition across participants of rsMEG variability for each frequency band. The frequency bands had a different number of independent components according to the MDL criterion (see text). In the top left and right corners of each topography are shown spatial correlations of each IC topography to the group mean and ageing topographies on sensor level, respectively (rGS and rAS, where GS stands for group effects on sensor level and AS stands for ageing effects on sensor level). Below each topographic representation is shown the individual IC loading value for each participant (blue point) plotted against their age (*x*‐axis). Regression line (red colour) and effect size (*r* value) added to plots where the ageing effect was significant at *P*‐value < 0.05. [Color figure can be viewed in the online issue, which is available at http://wileyonlinelibrary.com.]

**Figure 8:**
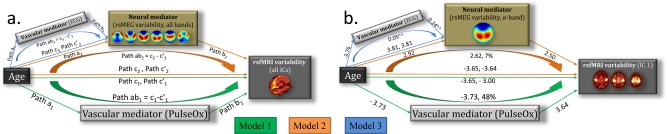
**a**) Generic path diagram representation of the mediation models used to determine whether vascular and neural variability differentially account for effects of age on rsfMRI variability (where each colour of arrow indicates a separate type of model), plus **b**) results from one specific model (using variability of time series in Alpha band for rsMEG as a mediator for IC1 of the RSFA). Two of the mediation models aimed to test whether either of the mediators (*M*) — (1) proxy measures of vascular health (i.e., HRV; Model 1, green paths) or (2) neural variability (i.e., variance of time series for standard frequency bands; Model 2, orange paths)‐explained a significant portion of the shared variance between the independent variable (*IV*, age) and the dependent variable (*DV*, rsfMRI variability). The third model (Model 3, blue paths) tested whether HRV mediated the effects of age on MEG neural activity at rest. The relationships between *IV‐M* and *IV‐DV* were characterized by path *a_i_* (*regression model: M = i_1_ + a_i_IV+e_1_*) and path ci (*DV = i_2_ + c_i_IV+e_2_*), respectively. Path bi described the relationship between *M‐DV*, while accounting for the effects of *IV*, path *c*'_*i*_ (*DV = i_3_ + b_i_M + c'_i_IV+e_3_*). Path abi indicated the presence of a significant difference between path *c_i_* and path *c'_i_*. The specific example shown in b) revealed that the ageing effects on rsfMRI variability (in most ICs, see Table 3) were significantly mediated (i.e., significant difference between path *c_1_* and path *c'_1_*, explaining 48% of the variance) by a summary measure of vascular health (Model 1, green paths). MEG variability in the alpha band mediated the ageing effects on RSFA loading values in IC1 (and IC5, see Table 4), explaining 7% of the variance (Model 2, orange paths), whereas the ageing effects on the MEG variability were not mediated by vascular health measures (Model 3, blue paths). Values beneath paths indicate the *Z*‐value of the test at a significance level *P* < 0.05. Percent values beneath paths *ab_i_* indicate the effect size calculated from the proportion between path *ab_i_* and path *c_i_*. [Color figure can be viewed in the online issue, which is available at http://wileyonlinelibrary.com.]

In terms of ageing, beta and gamma signal variability in frontal and temporal regions increased with age, whereas alpha band variability in visual areas decreased with age. Lower frequency bands (subdelta, delta and theta bands) showed age‐related increases in signal variability in peripheral sensors (Fig. 6).

##### Topography of rsMEG variability from ICA

From ICA of the rsMEG power in each frequency band, the optimal number of components according to the MDL criterion was 3, 3, 2, 4, 3, and 2 for subdelta, delta, theta, alpha, beta, and gamma bands, respectively. The topographies of the ICs for each band, and their relationships with age are summarized in Figure 7.

#### PCA analysis of HRV

PCA analysis estimated the first principal component (PC1) to explain approximately 65% of the variance across the three summary measures of HR, whether those measures were derived from the PulseOx recordings in the MRI scanner, or the ECG recordings in the MEG scanner. The absolute contribution to PC1 was similar across measures, varying from 0.39 to 0.67 (Table 2). As expected, PC1 from the PulseOx data (PC1_PulseOx_) was highly correlated with PC1 from the ECG data (PC1_ECG_), *r* = 0.741, *P* < 0.001.

**Table 2:** Total variance explained and coefficients of each principal component (PC) for the cardiac variables derived for both pulse oximetry and ECG data

	PulseOx PCA			ECG PCA		
	PC 1	PC 2	PC 3	PC 1	PC 2	PC 3
variance explained (%)	67.96	23.58	8.45	64.40	28.44	7.16
mean HR	−0.48	0.85	0.20	−0.39	0.90	0.18
LF‐HRV	0.60	0.49	−0.63	0.63	0.41	−0.66
HF‐HRV	0.64	0.19	0.75	0.67	0.15	0.73

These physiological recordings were used to derive mean HR, low‐frequency HRV (LF‐HRV) and high‐frequency HRV (HF‐HRV).

**Table 3:** Mediation between Age and RSFA components, by HRV (Model 1) and subdelta‐band MEG (Model 2)

	Paths																														
		*a*						*b*						*c'*						*c*						*ab*					
M	DV	β	*SE*	*Z*	*p*	*LLCI*	*ULCI*	β	*SE*	*Z*	*p*	*LLCI*	*ULCI*	β	*SE*	*Z*	*p*	*LLCI*	*ULCI*	β	*SE*	*Z*	*p*	*LLCI*	*ULCI*	β	*SE*	*Z*	*p*	*LLCI*	*ULCI*
HRV_PulseOx_	IC 1	−.492	0.046	−3.730	<.001	−.599	−.385	0.298	0.054	3.643	<.001	0.167	0.421	−.158	0.053	−3.000	0.003	−.287	−.034	−.305	0.051	−3.646	<.001	−.426	−.187	−.146	0.030	−3.725	<.001	−.219	−.080
	IC 3	−.492	0.046	−3.712	<.001	−.599	−.384	0.345	0.052	3.631	<.001	0.223	0.469	−.180	0.053	−3.292	0.001	−.304	−.056	−.350	0.050	−3.610	<.001	−.469	−.238	−.169	0.030	−3.705	<.001	−.246	−.106
	IC 5	−.492	0.046	−3.756	<.001	−.600	−.383	0.349	0.051	3.650	<.001	0.225	0.464	−.052	0.057	−.907	0.365	−.184	0.084	−.223	0.053	−3.743	<.001	−.347	−.100	−.172	0.030	−3.659	<.001	−.249	−.109
	IC 6	−.492	0.046	−3.744	<.001	−.595	−.382	0.130	0.054	2.504	0.012	0.002	0.255	−.466	0.053	−3.863	<.001	−.593	−.345	−.530	0.046	−3.834	<.001	−.638	−.422	−.064	0.028	−2.416	0.016	−.133	−.002
	IC 7	−.492	0.046	−3.712	<.001	−.599	−.384	0.206	0.051	3.594	<.001	0.082	0.321	−.013	0.052	−.324	0.746	−.131	0.112	−.115	0.051	−2.409	0.016	−.229	0.008	−.102	0.027	−3.689	<.001	−.169	−.042
	IC 8	−.492	0.047	−3.702	<.001	−.601	−.383	0.249	0.056	3.718	<.001	0.121	0.379	−.087	0.059	−1.493	0.135	−.225	0.048	−.209	0.053	−3.664	<.001	−.336	−.090	−.122	0.030	−3.657	<.001	−.197	−.059
	IC 11	−.492	0.046	−3.693	<.001	−.604	−.386	−.171	0.057	−3.135	0.002	−.310	−.043	.159	0.056	2.780	0.005	0.025	0.285	0.243	0.051	3.804	<.001	0.123	0.359	−.084	0.029	−3.313	.001	−.022	−.160
	IC 12	−.492	0.046	−3.707	<.001	−.600	−.384	0.216	0.062	3.541	<.001	0.080	0.365	.416	0.057	3.778	<.001	0.286	0.552	0.309	0.051	3.681	<.001	0.189	0.429	−.106	0.033	−3.464	.001	−.192	−.040
Sub‐δ‐band (.01–08 Hz)	IC 1	0.120	0.056	2.467	0.014	0.006	0.268	0.173	0.059	3.143	0.002	0.048	0.328	−.326	0.052	−3.574	<.001	−.455	−.212	−.305	0.051	−3.614	<.001	−.430	−.191	−.021	0.010	2.747	.006	−.003	.052
	IC 5	0.120	0.056	2.454	0.014	0.006	0.269	0.128	0.059	2.467	0.014	0.008	0.286	−.238	0.054	−3.668	<.001	−.367	−.113	−.223	0.053	−3.704	<.001	−.347	−.099	−.015	0.008	2.612	.009	−.002	−.043

The mediation regression Coefficients (β), Standard Errors (*SE*), *Z*‐scores, *P‐*values and 99% CIs (LLCI and ULCI) for paths *a*, *b*, *c*, *c'*, and *ab* (see Fig. 8). The independent variable (IV) and the dependent variable (DV) were age and RSFA independent component (IC) loading values, respectively, for tests with 99% CIs not including zero. The mediator (M) in Model 1 was HRV during resting state fMRI scan recorded with pulse oximetry, while in Model 2 was global rsMEG variability in beta band.

#### Mediation analysis

One of the main goals of the present study was to investigate the differential contribution of vascular and neural signals to RSFA. For the purpose, we conducted three mediation models using a product‐of‐coefficients approach (Hayes, 2013) to determine whether vascular and neural variability might differentially account for the observed effects of age on RSFA in our cohort, while controlling for handedness and gender.

##### Model 1: HRV mediating age differences in rsfMRI variability

The first mediation analysis addressed whether HRV mediated the effects of ageing on RSFA, where age, HRV (based on PC1_PulseOx_ loading values) and RSFA (based on age‐dependent PC loading values, see Fig. 5) were treated as the independent variable, the mediator and the dependent variable, respectively.

The mediation analysis provided estimates of the direct effect of age on HRV (See Fig. 8, path *a*_1_) and the direct effect of HRV on RSFA, while additionally controlling for age (path *b*_1_). The model also estimated the indirect effect of age on RSFA by way of HRV (path *ab*_1_) and the direct effect of age on RSFA (path *c'*_1_) when accounting for path *ab*_1_.

As can be seen from Table 3 and Figure 8 (green paths), age was associated with lower HRV (negative *a*), and participants with low HRV (irrespective of their age) showed differential expression of the spatial patterns for all ICs of RSFA (significant *path b*), except for IC2 and IC10. The indirect effect of age on RSFA through HRV was reliably negative according to bootstrap 99% CIs (path *ab*). There was evidence that the direct effect of age on RSFA, independent of HRV, was significantly reduced, that is, the absolute value of *path c'* was smaller than *path c*, indicating that HRV significantly mediated the ageing effects on RSFA IC loading values.

##### Model 2: Resting state MEG variability mediating age differences in RSFA

In a second set of analyses, we were interested in whether the effects of ageing on rsfMRI variability in neuronally meaningful areas (RSFA maps with spatial distribution within GM, that is, IC1, IC2, IC5, IC6, IC8, and IC10) were mediated by measures of neural variability. In this analysis age, rsMEG variability and rsfMRI variability were treated as the independent variable, the mediator and the dependent variable, respectively. As a summary measure of rsMEG variability, we used the grand mean of time‐series variability across all sensors for six frequency bands (subdelta, delta, theta, alpha, beta, and gamma), see Figure 8b.

Out of 36 mediation tests (six frequency bands x six ICs of RSFA maps over GM areas), only two exceeded the 99% CIs around zero. These were the models with rsMEG variability in the sub‐delta frequency band as a mediator and RSFA loading values in IC1 and IC5 as the dependent variable (Table 3). This model suggested that age was associated with increased rsMEG variability (positive *path a*), that participants with large rsMEG variability showed greater expression of the RSFA ICs (positive *path b*), that the indirect effect of age on RSFA through rsMEG variability was significantly positive (positive *path ab*), and that the negative effect of age on RSFA significantly increased after controlling for the indirect effects of rsMEG variability (the absolute value of *path c'* was larger than *path c*), that is, rsMEG variability was a significant suppressor, not mediator, of the ageing effects on RSFA.

One could argue however that global rsMEG variability across all sensors is not sensitive to detect local differences in rsMEG variability as a mediator of RSFA. For example, spatially distinct sensors might show effects of ageing in opposite directions (see, e.g., Fig. 7, *Alpha band‐* *IC3 and IC4*) or weak ageing effects in few sensors might be washed out by averaging with the remaining sensors (see, e.g., Fig. 6, ageing effects in alpha band). Therefore, we repeated Model 2 of the mediation analysis using the loading values of those 15 ICs of the rsMEG variability that correlated with age (see *Section Topography rsMEG variability using ICA*). This resulted in 90 mediation tests (15 rsMEG ICs × 6 ICs of RSFA), of which five tests satisfied all conditions for mediation/suppression according to 99% CIs (see Table 4). These were the models with rsMEG variability of alpha_ic2_, beta_ic1,ic2_ as a mediator and RSFA loading values in IC1 and IC5 as dependent variable. Similarly as for global rsMEG variability, all models suggested that age was associated with increased rsMEG variability, that participants with large rsMEG variability showed greater expression of the RSFA ICs, and that the negative effect of age on RSFA significantly increased after controlling for indirect effects of rsMEG variability, that is, rsMEG variability in alpha and beta band was a significant suppressor of the ageing effects on RSFA in sensory regions.

**Table 4:** Mediation between Age and RSFA components, by rsMEG variability (Model 2)

	Paths																																
				*a*						*b*						*c*′						*c*						*ab*					
	*M*		*DV*	β	*SE*	*Z*	*p*	*LLCI*	*ULCI*	β	*SE*	*Z*	*p*	*LLCI*	*ULCI*	β	*SE*	*Z*	*p*	*LLCI*	*ULCI*	β	*SE*	*Z*	*p*	*LLCI*	*ULCI*	β	*SE*	*Z*	*p*	*LLCI*	*ULCI*
	α‐band	IC2	IC1	0.168	0.057	2.918	0.004	0.037	0.301	0.133	0.051	2.502	0.012	0.013	0.252	−.330	0.053	−3.652	<.001	−.456	−.211	−.308	0.051	−3.646	<.001	−.431	−.192	0.022	0.012	2.615	0.009	0.002	0.061
		IC2	IC5	0.168	0.057	3.095	0.002	0.038	0.300	0.135	0.055	2.502	0.012	0.010	0.265	−.251	0.055	−3.726	<.001	−.379	−.126	−.228	0.053	−3.722	<.001	−.352	−.108	0.023	0.012	2.673	0.008	0.002	0.062
	β‐band	IC1	IC5	0.371	0.052	3.732	<.001	0.250	0.494	0.147	0.060	2.389	0.017	0.006	0.290	−.283	0.057	−3.699	<.001	−.414	−.153	−.228	0.053	−3.666	<.001	−.353	−.107	0.055	0.024	2.497	0.013	0.004	0.117
		IC2	IC1	0.365	0.051	3.625	<.001	0.246	0.481	0.124	0.050	2.467	0.014	0.006	0.234	−.353	0.054	−3.632	<.001	−.483	−.231	−.308	0.051	−3.625	<.001	−.432	−.194	0.045	0.020	2.558	0.011	0.004	0.096
		IC2	IC5	0.365	0.051	3.663	<.001	0.244	0.481	0.176	0.058	3.006	0.003	0.040	0.310	−.293	0.058	−3.767	<.001	−.429	−.159	−.228	0.054	−3.732	<.001	−.353	−.104	0.064	0.024	3.077	0.002	0.016	0.127

The Mediation regression Coefficients (β), Standard Errors (*SE*), *Z*‐scores, *P‐*values and 99% CIs (LLCI and ULCI) for paths *a*, *b*, *c*, *c'*, and *ab* (see Fig. 8). The independent variable (IV) and the dependent variable (DV) were age and RSFA independent component (IC) loading values, respectively, for tests with 99% CIs not including zero. The mediator (M) was the IC loading value of rsMEG variability in different frequency bands.

##### Model 3: HRV mediating age differences in rsMEG variability

In the final mediation model, we tested whether HRV mediated the effect of age on neural activity at rest (Fig. 8b, blue colour paths), separately for each frequency band. Here, the mediator was the HRV, estimated from the ECG data collected during the resting state MEG scan, while age and rsMEG variability (for both, grand mean over sensors and IC loadings for each frequency) were treated as independent and dependent variables, respectively. We did not find any evidence that the HRV mediated the age differences in rsMEG variability.

## DISCUSSION

The principal aim of this paper was to assess the use of RSFA (i.e., rsfMRI variability) as a scaling parameter in the analysis of the effects of age on task‐related BOLD‐fMRI activations. Our results demonstrated the importance of such scaling to identify the effects of age on brain function: without accounting for influence of RSFA, there appeared to be a significant age‐related decline in activation of the primary visual and auditory regions during a sensorimotor task, accompanied by an increase in ipsilateral primary motor cortex. After RSFA scaling, the effect of age in the visual areas was significantly attenuated, to the extent that it was no longer significant. The effect of age in the motor cortex remained significant. We confirmed the hypothesis that individual differences in RSFA were related to cardiovascular factors. In particular, mediation analyses revealed that age‐related associations of widespread RSFA decreases across the brain were mediated by an age‐related decline in vascular functions. Moreover, by comparing with neurophysiological MEG data from the same participants, we demonstrated that age‐related changes of neuronal variability did not mediate the age‐related changes in RSFA. There was one possible exception to the latter statement: a small proportion of variance attributed to age differences of RSFA in sensory areas was influenced by neuronal differences as measured by MEG variance in the beta band. However, this was a suppression rather than mediation of the Age‐RSFA relationship (as discussed below).

### Ageing Effects on BOLD Response to Audiovisual Stimulation and the Effects of Haemodynamic Scaling with RSFA

The BOLD responses to the sensorimotor task indicated an age‐related decrease of BOLD in visual and auditory cortices, in accord with previous studies (Buckner et al., 2000; Davis et al., 2008; Ross et al., 1997). However, after accounting for vascular differences with RSFA, the ageing effects in these regions was not significant (Handwerker et al., 2007; Liu et al., 2013; Riecker et al., 2003; Thomason et al., 2007). Importantly, not all age differences disappeared after controlling for RSFA—for example, the ipsilateral motor cortex remained significant, consistent with the results of other methods used to study ageing effects on the motor system (Boyke et al., 2008; Kannurpatti et al., 2011; Rowe et al., 2006; Scholz et al., 2009; Tigges et al., 1990).

### Spatial Distribution and Age Differences in RSFA

At a group level, RSFA maps followed the spatial pattern of brain regions characterized previously by high vascular reactivity (Di et al., 2012; Kalcher et al., 2013; Kannurpatti et al., 2011; Liu et al., 2013; Yezhuvath et al., 2009). Ageing had negative effects on RSFA in the regions with high RSFA, in line with findings from studies using other calibrating techniques (Chen et al., 2011; Kannurpatti et al., 2011; Liu et al., 2013; Lu et al., 2011). As RSFA is sensitive to age differences in vascular reactivity (validation discussed below), it suggests that future studies need to account for vascular effects to avoid misattribution of age‐related effects in task‐related BOLD signal to neural changes.

Since the effects of age on RSFA were not uniform, we examined the independent sources of variance of the spatial extent of RSFA. We identified two age‐dependent components following the spatial distribution of brain regions with vascular origin, such as large blood vessels that provide arterial supply and venous draining. These results are in agreement with previous findings demonstrating the effectiveness of ICA to successfully identify major blood vessels (Carroll et al., 2002). Another pair of components showed an age‐related increase of RSFA within the ventricles, the cerebral aqueduct and subarachnoid space, which might reflect age‐dependent differences in pulsatility of the brain within CSF spaces (Schmid Daners et al., 2012; Wåhlin et al., 2014).

The source of signal variability in another set of components characterized three distinct spatial distributions, the expression of which reduced as a function of age: (i) sensory areas (visual, auditory and somatosensory cortices), (ii) the cerebellum, and (iii) association areas (ventrolateral parietal cortex and anterior prefrontal cortex). These patterns of spatially distinct cortical areas might reflect segregation of cortical tissue composition, for example, delineation on the basis of cyto‐ or myelo‐ architectonic differences (Annese et al., 2004; Fukunaga et al., 2010; Geyer and Turner, 2013; Glasser and Van Essen, 2011). Cortical areas defined by cyto‐ and myelo‐ architecture often differ in other ways, such as vascular density (Duvernoy et al., 1981; Gardner, 2010; Guibert et al., 2012; Harrison et al., 2002; Lauwers et al., 2008; Zheng et al., 1991), neuron density (Beaulieu and Colonnier, 1989; Cahalane et al., 2012; Collins et al., 2010; Jespersen et al., 2010; Semendeferi et al., 2001; Young et al., 2013) and burden of vascular beta‐amyloid deposits (Rodrigue et al., 2012, 2009).

Since age‐related changes in the structure, vasodilatory capacity and other biomechanistic properties of cerebral blood vessels could lead to increased risk of vascular beta‐amyloid deposition, disrupted autoregulation and impaired vascular reactivity (Kalaria, 2010; Legge and Hachinski, 2010), it is possible that the sources of signal variability in these components reflect the organization of intracortical vascular territories (Gardner, 2010). Another possible source of signal that could in principle lead to the spatial pattern of age‐dependent RSFA components is the neuronal activity within resting state networks (Biswal et al., 1995; Fox et al., 2005), which we consider in the next sections.

### Cardiovascular Factors Mediate Age Effects on RSFA

Using mediation analyses, we found evidence that vascular measures can partly explain the ageing effects in all RSFA components, except for cerebellum and prefrontal cortex, that is, individuals with poor regulation of their vascular function showed widespread RSFA decreases across the brain. Individuals' vascular functions were measured by combining signals from mean HR, LF‐HRV, and HR‐HRV. Previous reports suggested that the aggregation of HRV‐based indices using PCA provides a compact summary of vascular function, while discarding redundancies in the high correlation between factors (Varadhan et al., 2009). Similar to ICA for RSFA and rsMEG variability, PCA minimises the statistical problem of multiple comparisons when testing for associations between RSFA, HRV, and rsMEG. In addition, we demonstrated the reliability of estimates of HRV signals by the high correlation between the first PC derived from pulseoximetry and the first PC derived from ECG, each acquired on separate occasions. Both measures detected decreased HRV as a function of age. These results show that age differences in RSFA reflect changes in the vascular integrity (Behzadi and Liu, 2005; Chang et al., 2009, 2013; Kannurpatti and Biswal, 2008; Kannurpatti et al., 2011; ShmueLi et al., 2007).

We did not observe evidence for HRV mediation on the ageing effects of RSFA in the areas of cerebellum, prefrontal cortex and CSF. One possible explanation is that HRV and RSFA might show low dependency in regions sensitive to susceptibility artefacts. For example, the cerebellum is in close proximity to large vessels of venous drainage, while BOLD signal in anterior parts of PFC is commonly associated with BOLD signal drop‐out. With respect to CSF, one would expect the effects of vasculature and CSF pulsatility to be independent from each other, so the lack of evidence in CSF areas for HRV mediation of the age‐dependent differences in RSFA signal is not surprising.

### rsMEG Variability Mediating Age Differences in RSFA

Our measure of neuronal variability was derived from rsMEG variability, which we captured by the variance of the time series in standard frequency bands, analogously to the BOLD measure of RSFA. ICA has shown that the spatial distribution of MEG variability is broadly consistent with that of BOLD signal variability (Brookes et al., 2011; Luckhoo et al., 2012).

The ageing effects we observed on rsMEG variability (across sensors or spatially independent components) are in agreement with previous reports using spectral power and signal complexity measures (Dustman et al., 1993, 1999; Gómez et al., 2013; McIntosh et al., 2013; Vlahou et al., 2014). Specifically, we observed age‐related changes in the spatial distribution of resting MEG signal variability, that is, beta and gamma signal variability increased over frontal and temporal sensors, whereas alpha band signal variability decreased over visual sensors. The age‐related increase in the signal variability of the higher frequency bands (beta and gamma) might be linked to less efficient GABAergic inhibition, which may lead to enhanced neuronal excitability (Schlee et al., 2012). In addition, delta and theta bands (MEG components delta_ic1_ and theta_ic2_) showed an age‐related decrease in tempo‐central regions (Vlahou et al., 2014) and an age‐related increase in visual areas (delta_ic2_ and theta_ic1_). We also observed an age‐related increase in the alpha band and a decrease in the beta band over superior temporo‐parietal sensors, which might suggest age‐related slowing of resting‐state oscillations not be restricted to alpha band (Dustman et al., 1993).

We investigated the degree to which RSFA reflects neural activity at rest, by comparing the relationship between RSFA and rsMEG variability. We found that rsMEG variability in beta and gamma bands (which increased as a function of age) contributed positively to the RSFA signal in sensory areas, that is, individuals with large neural variability showed large RSFA, an effect that remained even after accounting for the direct effect of ageing. This suggests that there is an effect of ageing on RSFA with a neural origin, which should increase with age, despite the observed overall age‐dependent decrease in RSFA. In other words, there might be two effects (neural and vascular) of ageing on RSFA in opposite directions, where an age‐dependent increase in RSFA of neural origin is concealed by an age‐dependent decrease in RSFA with vascular origin.

This dual effect of ageing on RSFA was confirmed in the mediation analysis, where we found no evidence that age‐related variance in neural activity (indexed by rsMEG variability) mediated positive age differences in RSFA across the whole brain. Instead, the effects of ageing on RSFA in sensory areas was suppressed, with a small portion of variance attributed to rsMEG variability in the alpha and beta band. In other words, accounting for the effects of rsMEG variability, the ageing effects on RSFA increased; or that, the suppression effect of rsMEG variability masked a small portion of the ageing effects on RSFA dominated by vascular modulatory origin in the sensory areas. As mentioned above, the contribution of neural activity to the RSFA might be as a result of functionally unspecific unregulated enhanced neuronal excitability within regions with increased rsMEG variability at rest as a result of less efficient GABAergic inhibition in ageing (Schlee et al., 2012). One explanation for the contribution of neural activity to RSFA in the auditory areas might be changing sensitivity to background scanner noise (Fabiani et al., 2006; Gaab et al., 2007; Grady et al., 2011; Moran et al., 2014; Scarff et al., 2004). It is important to remember that we derived the RSFA from resting state fMRI time series. In contrast, Garrett et al. (Garrett et al., 2010, 2011, 2012) measured the BOLD signal variance during blocks of different cognitive states. This active state fluctuation predicted cognitive performance but declined with age, perhaps due to a reduced ability to efficiently process unexpected external stimuli (Grady and Garrett, 2013).

Together, our data suggest that the age‐dependent change in neural activity at rest is small, offsetting a small proportion of the RSFA signal in sensory cortex. As the effects were in the opposite direction to the overall ageing effects on RSFA (driven by vascular differences), we propose that scaling task‐induced BOLD signal by RSFA can still account for differences in vascular function, without removing the principle signals of neuronal origin.

### Limitations

Although MEG offers a direct measure of neural activity, there are practical issues when trying to relate MEG to fMRI signals on intra‐ and interindividual basis. For instance, one reason why we did not observe rsMEG variability mediating the effects of age on RSFA might be due to MEG's relative insensitivity to signals from radially oriented sources, and from deep cortical and subcortical regions (Ahlfors et al., 2010; Cohen and Cuffin, 1991). Future use of EEG, perhaps in combination with MEG, might be more sensitive to interindividual differences in radial and deep sources of neural activity (Hämäläinen et al., 1993). It is possible that the variability of neural signals after localising MEG data (i.e., source estimation) might improve sensitivity in identifying their mediating effects on RSFA. However, source localization is a linear unmixing of sensor signals (analogously to our use of ICA), so does not produce more information. This means that it is unlikely to increase the chance of finding a statistically significant mediation. In addition, the forward problem for source localization of ageing effects presents a difficulty, since head modelling and dipole fitting may be confounded by grey matter atrophy and head movement, leading to deviations from the true lead fields (Sarvas, 1987) and volume conduction (Van den Broek et al., 1998), which can result in erroneous source reconstruction (Hillebrand and Barnes, 2011). Therefore, we adopted group ICA on sensor level, rather than source level, to decompose rsMEG variability into spatially independent sources. Future studies using validated approaches for confound‐free MEG/EEG source localization would be valuable to confirm our findings.

Another potential methodological limitation in the current design was that MEG and fMRI scans were acquired on two separate occasions. Subtle changes in the resting conditions (Bianciardi et al., 2009), performance on preceding cognitive tasks (Barnes et al., 2009), or drowsiness (Horovitz et al., 2009) could have affected the resting functional connectivity. For example, the MRI data were acquired while supine, whereas the MEG data were acquired while seated. Despite this concern, there is empirical evidence for high test–retest reliability over periods of 6 months, at elast for intrinsic activity (Zuo et al., 2010a) and functional connectivity (Braun et al., 2012; Guo et al., 2012; Song et al., 2012; Zuo et al., 2010b). Moreover, our design matched experimental conditions between sessions and the interval between MEG and fMRI did not vary as a function of age. Future studies using simultaneously acquired EEG and fMRI would be valuable to confirm our findings (Mullinger et al., 2013).

One other point pertains to physiological factors such as respiration and end‐tidal CO_2,_ which also contribute significant proportions of the rsBOLD signal variance (Chang et al., 2009; Glover et al., 2000); over and beyond HRV (Golestani et al., 2015). Some of these studies provided methods for removing signals related to these physiological factors from further analysis (Chang et al., 2009; Golestani et al., 2015). One could take advantage of these approaches to extract physiologically specific rsBOLD signals, which could be further used for scaling task‐based fMRI data. This was not feasible in the current study given that no end‐tidal CO_2_ and respiratory recording were available, but would be valuable to confirm this in future work.

The sample in our study, unlike most cross‐sectional neurocognitive studies, was from a population‐based cohort that had a uniformly distributed age range extending from early adulthood (18–28 years) to late age (78–88 years). Although the population sampling method for this study sought to minimize age‐based cohort effects, future studies would be strengthened by longitudinal analysis.

## CONCLUSIONS

We propose that RSFA can be used as a robust scaling factor to control for vascular differences in task‐induced BOLD signal, particularly in the context of fMRI studies of ageing. RSFA is, therefore, a suitable alternative to other calibrating techniques (such as breath‐holding and hyper/hypocapnia), where hypercapnic challenge would be inappropriate (e.g., for older or frail participants). Without such scaling methods, fMRI studies of the effects of age on cognition may misinterpret effects of age as neurocognitive, rather than neurovascular, phenomena.
